# Magnetic, Phonon, and Optical Properties of Pure and Doped Ba_2_FeReO_6_ and Sr_2_CrReO_6_—Bulk Materials and Nanoparticles

**DOI:** 10.3390/ma18061367

**Published:** 2025-03-19

**Authors:** Angel T. Apostolov, Iliana N. Apostolova, Julia M. Wesselinowa

**Affiliations:** 1 Department of Physics, Faculty of Hydrotechnics, University of Architecture, Civil Engineering and Geodesy, 1046 Sofia, Bulgaria; angelapos@abv.bg; 2 Faculty of Forest Industry, University of Forestry, 1756 Sofia, Bulgaria; inaapos@abv.bg; 3 Faculty of Physics, Sofia University “St. Kliment Ohridski”, J. Bouchier Blvd. 5, 1164 Sofia, Bulgaria

**Keywords:** Ba_2_FeReO_6_ and Sr_2_CrReO_6_, magnetization, phonon energy, microscopic model, Green’s function theory

## Abstract

On the basis of a microscopic model and employing Green’s function technique, the effects of temperature, size, and ion doping on the magnetization and phonon energy of the A_1*g*_ mode in double perovskites Ba_2_FeReO_6_ and Sr_2_CrReO_6_—both in bulk and nanoscale samples—are investigated for the first time. The Curie temperature *T_C_* and magnetization *M* decrease as nanoparticle size is reduced. Doping with rare-earth ions such as Sm, Nd, or La at the Ba or Sr sites further reduces *M*. This behavior originates from the compressive strain induced by the smaller ionic radii of the dopant ions compared to the host ions. As a result, the antiferromagnetic superexchange interaction between Fe or Cr and Re ions is enhanced, along with an increase in the magnetic moment of the Re ion. The dependence of the band gap energy of Sr_2_CrReO_6_ on temperature, size, and doping is also studied. Near the magnetic-phase-transition temperature *T_C_*, anomalies in phonon energy and damping indicate strong spin–phonon coupling. The theoretical calculations show good qualitative agreement with experimental data.

## 1. Introduction

In recent years, double-perovskite compounds of the form X_2_ T′T″O_6_ (where X represents an alkaline-earth metal and T′ and T″ correspond to transition metal cations with 3d, 4d, or 5d orbitals) have attracted significant research interest due to their remarkable physical properties [1,2]. These oxides possess relatively simple crystalline structures, making them ideal for studying fundamental magnetic interactions in solids. Double perovskites typically exhibit a transition from ferromagnetism to paramagnetism at a Curie temperature *T_C_* that exceeds room temperature, making them promising candidates for spintronic applications [1,2]. This magnetic transition is driven by the superexchange interaction between T′^3+^ and T″^5+^ ions. Certain compounds, such as X_2_ T′ReO_6_ (X = Sr, Ba; T′ = Fe, Cr), have been extensively investigated due to their spin-polarized conduction electrons, which originate from a half-metallic ferrimagnetic ground state and exhibit magnetic ordering transitions at room temperature [3,4,5]. Some double-perovskite materials, such as Ba_2_ FeReO_6_ (BFRO) and Sr_2_CrReO_6_ (SCRO), have garnered significant attention due to their intriguing properties and various applications [6,7,8,9,10]. The nature of T-site cations and their interactions is responsible for the magnetic behavior and magnetoresistance which assist double perovskites in spintronic applications with the manipulation of the spin of electrons for information processing. The efficient ability of band gap tuning and semiconducting behavior along with thermal stability makes the material suitable for optoelectronic applications like photodetectors, photovoltaics, solar cells etc. In addition, double perovskites exhibit the ability to absorb light over a broad range of the spectrum which makes them potential candidates for the usage in light-emitting devices. These compounds exhibit a tetragonal lattice at low temperatures and transform into a cubic lattice at room temperature, with highly ordered Fe (or Cr) and Re sublattices. They are proposed to be ferrimagnetic half-metals, possessing high magnetic-phase-transition temperatures $T_{C}$ of 303 K for BFRO and 625 K for SCRO.

The magnetic properties of BFRO and SCRO are studied extensively experimentally [11,12,13,14]. Kato et al. [11] found that SCRO is a metallic ferromagnet with a high Curie temperature of $T_{C}$ = 635 K, whereas Ca_2_CrReO_6_ is a ferromagnetic Mott insulator with $T_{C}$ = 360 K. Sikora et al. [12] observed a considerable orbital magnetic moment in the double perovskites X_2_FeReO_6_ (⁠X = Ba, Sr, and Ca) using X-ray magnetic circular dichroism at the Re L_2.3_ edges. Moreover, the spin moment of Re ion scales with Curie temperature, the most relevant property in spin electronics applications of the compounds studied. Yang et al. [13] considered the magnetoelastic coupling and microstructure dynamics associated with spin–orbit coupling in BFRO which is effectively synonymous with spin–lattice coupling if the spontaneous strain arises essentially as a consequence of changes in electronic structure that are strongly dependent on spin state.

BFRO and SCRO are theoretically investigated using ab initio calculations and Monte Carlo simulations [15,16,17,18]. Using a full-potential linear muffin-tin-orbital density functional method, Vait et al. [15] observed that double perovskites SCRO and BFRO are half-metallic, but when including the spin–orbit coupling, the 5d transition Re ions exhibit substantial unquenched orbital magnetic moments, resulting in a significant increase in the total magnetic moment. Melo et al. [16] studied the magnetic and electronic properties of double perovskites X_2_FeReO_6_ with X = Ba and Ca using ab initio methods based on the density functional theory. They have shown that, when changing Ba with Ca, the system undergoes a metal–insulator transition. Kerrai et al. [17,18] investigated the electronic, magnetic, and optical properties of BFRO using ab initio and Monte Carlo studies. The density of states and band structures were calculated using the GGA technique.

The origin of magnetism in these double perovskites is attributed to the interaction between the mixed 3d/5d transition metal ions at the T′/T″ sites, as well as the strong interaction between the localized 3d electrons and the more delocalized 5d electrons, which exhibit significant spin–orbit coupling. Studies on nanosized BFRO and SCRO powders have been reported in [19,20,21,22,23], indicating a reduction in spontaneous magnetization compared to their bulk counterparts. Fuoco et al. [20] measured the magnetic properties as a function of magnetic field strength and analyzed them with respect to the extent of T′/T″ site ordering and resultant particle sizes for SCRO and BFRO. Additionally, the effects of La, Nd, and Sm substitution in SCRO have been investigated by Michalik et al. [24,25], while Y doping in SCRO has been examined by Orlando et al. [26] and La doping in BFRO has been examined by Wang et al. [27].

Garcia-Flores et al. [28] examined the temperature dependence of the frequency and line width of the A_1*g*_, E_*g*_, and F_2*g*_ modes in BFRO, as well as the A_1*g*_ mode in SCRO. There is a remarkable hardening of the normal mode below the Curie temperature $T_{C}$. Moreover, the A_1*g*_ symmetric stretching phonon mode shows a kink around $T_{C}$ due to the spin–electron–phonon interaction, whereas the $E_{g}$ anti-symmetric stretching mode does not show such an anomaly at $T_{C}$.

Band gaps are crucial for the optical and electrical characteristics of semiconductor compounds. The band gap $E_{g}$ in BFRO and SCRO is studied in [15,20,29,30]. The calculated band gap of SCRO is 0.22 eV [20], which is close to the experimental value of 0.21 eV [30]. Vaitheeswaran et al. [15] reported that for BFRO, the value of $E_{g}$ is 0.7 eV.

The majority of theoretical papers that studied the magnetic properties of BFRO and SCRO are based on the density functional theory (DFT) [15,16,17,18]. DFT is a very powerful tool for investigating many-body problems. However, DFT is mostly concerned with ground-state properties at zero temperature. In our approach, we are able to cover the whole temperature regime. It is a finite temperature analysis and includes the entire excitation spectrum. In particular, the method allows us to study the total phase diagram, which is based on the different excitation energies realized in the system. The disadvantage of our approach lies in the consideration of collective properties from the beginning. Our basic quantities are not the naked electrons but effective spins of the underlying quasi-particles. However, with DFT, all parameters of the system can—at least in principle–be calculated, so we are forced to use additional models to determine these parameters. We are convinced that both approaches, DFT and Green’s function method, are appropriate and, to a certain extent, can be alternatives for describing many-body systems.

This paper presents, for the first time, an investigation of the magnetization, band gap, and phonon energy of pure and ion-doped BFRO and SCRO (for both bulk and nanoscale samples) using a microscopic model and Green’s function theory [31].

## 2. The Model

Considering the spin–phonon interaction observed in both pure and rare-earth (RE)-doped BFRO and SCRO [28], the Hamiltonian governing the magnetic and phononic properties of these materials can be expressed as follows:

(1) $$H=H_{m}+H_{ph}+H_{m-ph}.$$

$H_{m}$ describes the magnetic subsystem as $H_{m}=H_{sp}+H_{el}+H_{sp-el}$ for BFRO and SCRO (in the latter, the Fe ion is replaced with the Cr ion). $H_{sp}$ is the modified Heisenberg model:

(2) $$\begin{array}{ccc} H_{sp} & = & -\sum_{ij}J_{ij}^{Fe-Fe}S_{i}^{Fe}\cdot S_{j}^{Fe}-\sum_{ij}J_{ij}^{Re-Re}S_{i}^{Re}\cdot S_{j}^{Re}\\ & - & \sum_{ij}\left(1-x\right)J_{ij}^{Fe-Re}S_{i}^{Fe}\cdot S_{j}^{Re}-\sum_{ij}xJ_{ij,d}^{Fe-Re}S_{i}^{Fe}\cdot S_{j}^{Re}\\ & - & \sum_{i}D_{i}^{Fe}{\left(S_{i}^{Fe}\right)}^{2}-\sum_{i}D_{i}^{Re}{\left(S_{i}^{Re}\right)}^{2}-g\mu_{B}h\sum_{ij}\left(S_{i}^{Fe}+S_{i}^{Re}\right). \end{array}$$

The first two terms are interactions within Fe and Re layers, respectively. The third term refers to inter-layer interactions. The exchange coupling interaction constants are denoted as $J_{ij}$, while $D_{i}$ represents the single-site anisotropy parameters. The applied external magnetic field is given by *h*, and the RE doping concentration varies within the following interval: *x* = 0–0.2. In BFRO, the spins of the Fe^3+^ and Re^5+^ ions are *S* = 5/2 (3d^5^ and *S* = 1 (5d^2^, respectively, with the net magnetic moments of 3.70 and −0.86 $\mu_{B}$. These ions interact antiferromagnetically via oxygen (superexchange interaction). Direct interactions between nearest-neighbor Fe-Fe or Re-Re pairs are absent.

In nanoscale samples, due to the increasing influence of the surface (as the surface-to-volume ratio increases) and the structural changes that occur in the compounds upon substitutional doping, their magnetic properties are modified. In our model, we take into account these facts through a simplified approximation by introducing distinct coupling parameters, $J_{s}$ and $J_{d}$, representing the interactions of surface spins and doping spins, respectively, in contrast to the bulk exchange coupling *J*. Depending on the strain induced by the doping ions, $J_{d}$ may either be larger or smaller than *J*. This approach is justified, as the exchange interaction parameter $J_{ij}\equiv J\left(r_{i}-r_{j}\right)$ is inherently dependent on the inter-spin distances and the lattice parameters. Consequently, variations in lattice symmetry and the coordination number of next-nearest neighbors influence the interaction strength.

$H_{el}$ determines the possibility for a conducting electron to perform hops between localized spin states without changing the direction of its spin:

(3) $$H_{el}=\sum_{ij\sigma }t_{ij}c_{i\sigma }^{+}c_{j\sigma },$$

where $t_{ij}$ defines the probability for the conducting electron to hop, i.e., the so-called hopping integral, as $c_{i\sigma }^{+}$ and $c_{i\sigma }$ are the Fermi operators of creation and annihilation for an electron at the *i*-th site, respectively.

$H_{sp-el}$ represents the so-called s–d interaction, which accounts for the pairing of localized spins and conducting electrons, and is given by the following form:

(4) $$H_{sp-el}=-\sum_{i}I_{i}S_{i}s_{i},$$

where *I* is the constant of the intra-atomic interaction, $S_{i}$ is the spin operator of the localized electron, and $s_{i}$ is the spin operator of the conducting electron at the *i*-th site. The latter can be expressed using the Fermi operators of creation and annihilation as follows: $s_{i}^{+}=c_{i+}^{+}c_{i-}$, $s_{i}^{z}=\left(c_{i+}^{+}c_{i+}-c_{i-}^{+}c_{i-}\right)/2$.

For numerical calculations of the band gap $E_{g}$, we will use the following equation:

(5) $$E_{g}=\epsilon^{+}\left(k=0\right)-\epsilon^{-}\left(k=k_{\sigma }\right),$$

where $\epsilon^{+}\left(k=0\right)$ and $\epsilon^{-}\left(k=k_{\sigma }\right)$ represent the energy at the top of the valence band and the energy at the bottom of the conduction band, respectively, where the minimum energy level of the conduction band aligns with the maximum energy level of the valence band at the same momentum. These electron energies are determined by the following expression:

(6) $$\epsilon_{ij}^{\pm }=\epsilon_{ij}-\frac{\sigma }{2}IM.$$

The electronic energies are determined by the poles of the single-particle Green function $g_{ij\sigma }=\ll c_{i\sigma };c_{j\sigma }^{+}\gg$, where $\epsilon_{ij}$ is the energy of the band electrons in the paramagnetic region. *M* is the magnetization of the sample.

$H_{ph}$ describes the lattice vibrations, considering the anharmonicity of the lattice (third- and fourth-order phonon–phonon interactions):

(7) $$\begin{array}{ccc} H_{ph} & = & \frac{1}{2!}\sum_{i}\omega_{0i}a_{i}a_{i}^{+}+\frac{1}{3!}\sum_{i,j,r}B\left(i,j,r\right)Q_{i}Q_{j}Q_{r}\\ & + & \frac{1}{4!}\sum_{i,j,r,s}A\left(i,j,r,s\right)Q_{i}Q_{j}Q_{r}Q_{s}, \end{array}$$

where $Q_{i}$ and $\omega_{0i}$ represent the normal coordinate and frequency of the lattice mode, respectively. The vibrational normal coordinate $Q_{i}$ can be written in terms of the phonon creation $a^{+}$ and annihilation *a* operators:

(8) $$Q_{i}={\left(2\omega_{0i}\right)}^{-1/2}\left(a_{i}+a_{i}^{+}\right).$$

As emphasized in the Introduction, the presence of the anomalous temperature behavior of phonon modes in these materials is evidence of strong spin–phonon interactions. The influence of spin ordering on the phonon energies and damping is accounted for with the following Hamiltonian:

(9) $$H_{m-ph}=\frac{1}{2}\sum_{i,j,k}F\left(i,j,k\right)Q_{i}S_{j}^{zFe}S_{k}^{zRe}-\frac{1}{4}\sum_{i,j,r,s}R\left(i,j,r,s\right)Q_{i}Q_{j}S_{r}^{zFe}S_{s}^{zRe}+h.c.$$

*F* and *R* represent the coupling constants of phonons to spin excitations in the first and second order, respectively. Theoretically, the spin–phonon interaction arises due to the strong dependence of the exchange interaction on polar lattice displacements. This leads to a quadratic dependence of the phonon energy on the magnetization [32], which manifests as an anomalous temperature behavior of the phonon frequency (hardening or softening with decreasing temperature and a kink at the temperature of the magnetic phase transition $T_{C}$). In our case, if this behavior is a consequence of the modulation of the exchange interaction parameter $J^{Cr(Fe)-Re}$ between two nearest-neighboring Cr(Fe)-Re atoms, such anomalies will be observed in all polar modes. From the experimental results of Garcia-Flores et al. [28], it is evident that this is the case only for the $A_{1g}$ mode but not for the $E_{g}$ mode. In [10], based on inelastic electron scattering experiments, it is shown that spin–phonon interactions are a consequence of strong spin–orbit coupling. This necessitates utilizing the renormalization of the lattice mode through its coupling with local spin moments. From a theoretical perspective, this is described by Equation (18). Formally, this implies accounting for the spin deviation at a given site caused by lattice vibrations. This describes the behavior of the $A_{1g}$ mode as a result of the collective excitation of charge, spin, and lattice degrees of freedom.

The magnetization in the ferromagnetic region for double perovskites is calculated from the following expression:

(10) $$M=\;|M^{A}-M^{B}|$$

with A = Fe(Cr) and B = Re. $M^{A,B}$ for arbitrary spin *S* is given by the following:

(11) $$\begin{array}{ccc} M^{A,B} & = & \langle S^{zA,B}\rangle =\frac{1}{N^{2}}\sum_{i,j}[\left(S^{A,B}+0.5\right)\coth\left[\left(S^{A,B}+0.5\right)\beta E_{ij}^{A,B}\right]\\ & - & 0.5\coth(0.5\beta E_{ij}^{A,B})], \end{array}$$

where 〈…〉 is the thermodynamic mean value.

The spin-wave energy $E_{ij}^{A,B}$ is calculated from the 2 × 2 matrix Green function as follows:

(12) $$G_{ij}^{A,B}\left(t\right)=\;\ll S_{i}^{+A,B}\left(t\right);S_{j}^{-A,B}\gg$$

This is calculated using the method of Tserkovnikov [33] presented shortly with Equations (13–16) taking into account the transverse correlation functions $\langle S_{i}^{-}S_{j}^{+}\rangle$ and decoupling the longitudinal ones $\langle S_{i}^{z}S_{j}^{z}\rangle \to \langle S_{i}^{z}\rangle \langle S_{j}^{z}\rangle$. The exchange interaction constant *J* is renormalized due to the spin–phonon interactions and is now temperature-dependent $J_{eff}=J+2F^{2}/\left(\omega_{0}-MR\right)$.

After performing a formal integration of the equation of motion for the delayed two-time Green function, the solution can be determined.

(13) $$G_{ij}\left(t\right)=\langle \langle S_{i}^{+}\left(t\right);S_{j}^{-}\rangle \rangle$$

The following can be obtained:

(14) $$G_{ij}\left(t\right)=-i\theta \left(t\right)\langle \left[S_{i}^{+};S_{j}^{-}\right]\rangle \exp\left(-iE_{ij}\left(t\right)t\right),$$

with θ(t) = 1 for t>0, and θ(t) = 0 for t<0,

(15) $$\begin{array}{ccc} E_{ij}\left(t\right)=E_{ij} & - & \frac{i}{t}\int_{0}^{t}dt^{\prime }t^{\prime }\left(\frac{\langle \left[j_{i}\left(t\right);j_{j}^{+}\left(t^{\prime }\right)\right]\rangle }{\langle \left[S_{i}^{+}\left(t\right);S_{j}^{-}\left(t^{\prime }\right)\right]\rangle }\right.\\ & - & \left.\frac{\langle \left[j_{i}\left(t\right);S_{j}^{-}\left(t^{\prime }\right)\right]\rangle \langle \left[S_{i}^{+}\left(t\right);j_{j}^{+}\left(t^{\prime }\right)\right]\rangle }{{\langle \left[S_{i}^{+}\left(t\right);S_{j}^{-}\left(t^{\prime }\right)\right]\rangle }^{2}}\right). \end{array}$$

Here, $j_{i}\left(t\right)=\langle \left[S_{i}^{+}\left(t\right),H_{interaction}\right]\rangle$. $E_{ij}$ is the excitation energy beyond the random phase approximation taking into account all correlation functions:

(16) $$E_{ij}=\frac{\langle \left[\left[S_{i}^{+},H\right];S_{j}^{-}\right]\rangle }{\langle \left[S_{i}^{+};S_{j}^{-}\right]\rangle }$$

From the time-dependent term in Equation (15), the damping can be calculated.

The renormalized phonon energy is calculated from the following phonon Green function:

(17) $$G_{ij}\left(t\right)=\langle \langle a_{i}\left(t\right);a_{j}^{+}\rangle \rangle$$

This equationuses the method of Tserkovnikov [33]. This approach allows us to go beyond the random phase approximation considering phonon correlation functions $\bar{N}=\langle a^{+}a^{-}\rangle$. The latter is determined with the help of the Spectral Theorem.

Following this procedure for the energy of the spin-dependent lattice modes, we obtain the following:

(18) $$\begin{array}{ccc} \omega {\left(k\right)}^{2} & = & \omega_{0}^{2}-2\omega_{0}\frac{1}{N}\sum_{q}(R\left(k,q\right)\langle S^{zFe}\rangle \langle S^{zRe}\rangle \\ & - & \frac{1}{2N}\sum_{q}A\left(k,q\right)\left(2\bar{N}\left(q\right)+1\right)-B\left(k\right)\langle Q_{k}\rangle ), \end{array}$$

with

(19) $$\langle Q_{k}\rangle =\frac{F_{k}\langle S^{zFe}\rangle \langle S^{zRe}\rangle -\frac{1}{N}\sum_{q}B_{k,q}\left(2{\bar{N}}_{q}+1\right)}{\omega_{0}-R_{k}\langle S^{zFe}\rangle \langle S^{zRe}\rangle +\frac{1}{N}\sum_{q}A_{k,q}\left(2{\bar{N}}_{q}+1\right)}.$$

The phonon damping is also evaluated taking into account anharmonic spin–phonon and phonon–phonon interactions.

## 3. Numerical Results and Discussion

The calculations are made on the basis of a numerical calculation program developed by the authors based on the JAVA software product and a self-consistent iterative procedure. The starting parameters for the iterations are the model parameters given below at *T* = 0 K.

The numerical calculations are performed based on the following model parameters: $J^{Fe-Fe}$ = 7.69 meV [17]; $J^{Re-Re}$ = 15.34 meV [17]; $J^{Fe-Re}$ = −14.05 meV [17]; $D^{Re}$ = −0.0036 meV [17]; $D^{Fe}$ = −0.0013 meV [17]; *I* = 0.2 eV; *F* = 21 cm^−1^; *R* = −19 cm^−1^; *A* = 6.6 cm^−1^; and *B* = −2.9 cm^−1^. The phonon–phonon interaction constants *A* and *B* are determined from the Raman spectra for temperatures above the Curie temperature $T_{C}$ (where the terms with *R* and *F* vanish), whereas the spin–phonon interaction constants *F* and *R* arise at very low temperatures, taking two values at two different temperatures from the Raman phonon energy and solving the system of two equations with two unknown parameters.

### 3.1. Temperature and Size Dependence of the Magnetization in BFRO and SCRO

**Figure 1:**
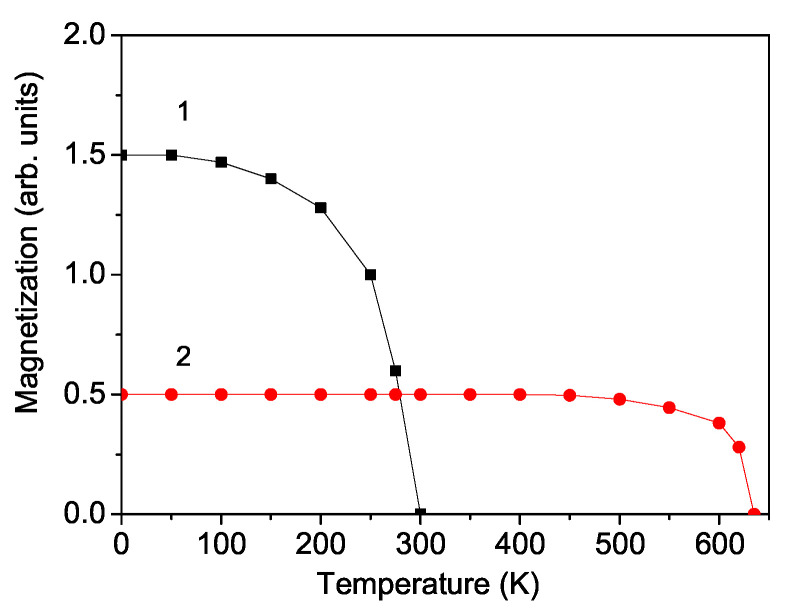
Temperature dependence of the magnetization for bulk BFRO (1) and SCRO (2).

We will firstly consider the magnetic properties of bulk BFRO and SCRO. From Equation (10), the magnetization *M* as function of temperature for these compounds is calculated, which is presented in Figure 1. The magnetization is reduced with increasing temperature and reaches zero at the Curie temperature of $T_{C}$ = 300 K for BFRO and at $T_{C}$ = 635 K for SCRO. These findings align well with experimental data reported by multiple authors [1,8,11,17]. Additionally, the coercive field $H_{c}$ decreases almost linearly with temperature as expected.

**Figure 2:**
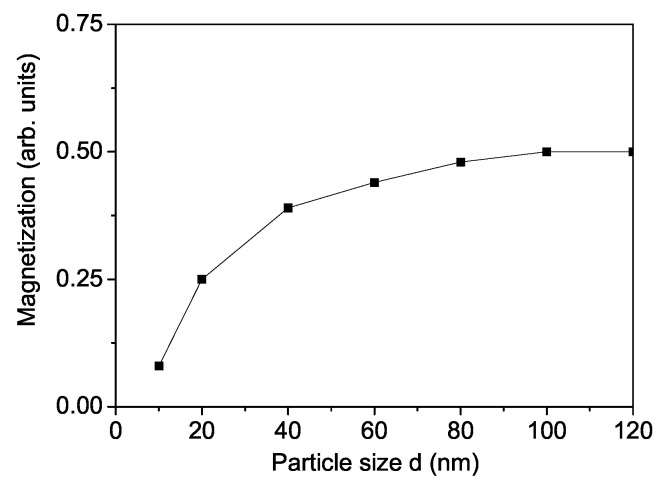
Particle size dependence of the magnetization for SCRO.

**Figure 3:**
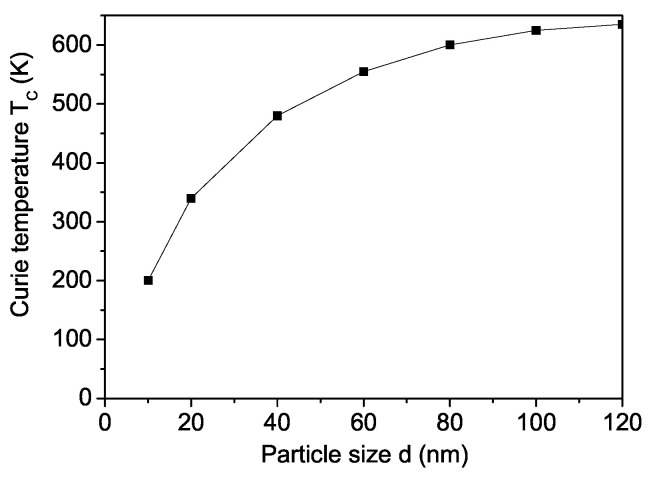
Particle size dependence of the Curie temperature $T_{C}$ for SCRO.

A nanoparticle (NP) is defined by setting the origin at a specific spin at its center and incorporating all surrounding spins within concentric shells. These shells are indexed as n = 1, …, N, where n = 1 corresponds to the central spin and n = N represents the outermost shell at the particle’s surface. The NP exhibits icosahedral symmetry. Due to surface disorder, defects, and uncompensated spins, both the magnetization *M* and the Curie temperature $T_{C}$ decrease as the NP size *d* is reduced. This effect is demonstrated for SCRO NPs in Figure 2 and Figure 3 for $J_{s}=0.8J$ for the ferromagnetic interaction constant, and $J_{s}=-0.8J$ for the antiferromagnetic constants. This relation refers to all interaction constants. It should be emphasized that Figure 2 shows that with a decrease in the surface-to-volume ratio, the magnetic properties of the nanoscale samples change drastically. This effect is ”amplified“ by the fact that we use an icosahedral particle. The saturation observed with increasing size is explained by the fact that the above-mentioned ratio becomes negligible and the surface has little influence on the properties of bulk materials which is proof that modeling the magnetic properties of the surface with $J_{s}<J$ is adequate. Concerning Figure 3, it should be noted that with decreasing NP sizes, the thermal fluctuations increase and they destroy the magnetic ordering. Therefore, at a certain critical size, the particle passes into a super-paramagnetic state and magnetization is not observed. A similar reduction in *M* and $T_{C}$ is observed by Lin et al. [19] and Fuoco et al. [20]. Lin et al. [19] obtained a Curie temperature of 350 K for NPs with a diameter of 20 nm, which closely matches our calculated value of 340 K for *d* = 20 nm. This very good agreement provides evidence that our model accurately captures the magnetic properties of SCRO. A comparable decrease in *M* and $T_{C}$ is also observed for BFRO NPs.

### 3.2. Ion Doping Dependence of the Magnetic Properties in BFRO and SCRO

Regarding the doping process, there are three factors that determine the change in the magnetic properties of the compounds under consideration:

(1) The difference in ionic radii creates compressive or tensile processes in the lattice.
(2) The difference in the degree of ionization between the substituted and substituting ions leads to the occurrence of anion oxygen vacancies to ensure charge neutrality of the compounds.
(3) The difference in single-ion anisotropy between substituted and substituting ions.

In the case of NPs, the surface effects should be added, including uncompensated bonds and disturbed number of nearest neighbors. All this leads to a complex change in the interactions at the microscopic level, which we model by changing the exchange interactions because the latter is most sensitive to changes in the distance between the magnetic ions. Moreover, the magneto-crystalline anisotropy is also very sensitive to changes in the intra-crystalline field during the doping process. In our opinion, from these factors, the exchange interactions as well as the magneto-crystalline anisotropy are the two key microscopic parameters, the change in which has a significant impact on the magnetic properties of the compounds under consideration. Insofar as the magnetically ordered states are essential, we consider that the lattice is tetragonal and the changes in the lattice parameters are taken into account by changing the exchange interactions (by introducing $J_{d}$ and $J_{s}$ in the NPs). As shown, the numerical results prove the adequacy of the model.

**Figure 4:**
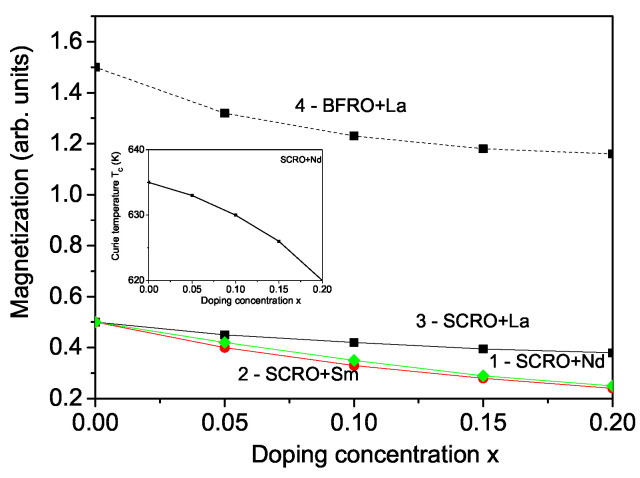
Doping ion concentration dependence of the magnetization for SCRO, at *T* = 10 K, for different dopants: Nd (1), Sm (2), and La (3), as well as for La-doped BFRO (4). Inset: Curie temperature $T_{C}$ as a function of the Nd doping concentration *x* in SCRO.

Next, we will study the influence of the substitution of different trivalent RE ions at the divalent Sr^2+^ site in SCRO, such as Nd^3+^, Sm^3+^, and La^3+^ on the spontaneous magnetization *M* and the Curie temperature $T_{C}$. The substitution changes the properties of these materials which could be applied for different applications. It should be noted that when ions with different degrees of ionization are substituted, anionic oxygen vacancies arise due to the requirement to maintain charge neutrality in the crystal [21,34]. This leads to changes in the internal crystal field and alters the angle in Cr(Fe)-O-Re. This angle will decrease compared to the optimal 180° angle that ensures antiferromagnetic ordering between the two neighboring magnetic ions. The magnetic moments of Nd, Sm, and La align parallel to those of Fe but antiparallel to the Re ions. Due to the differences in the ionic radii between the RE ions and the host ones, a strain is induced, altering the interaction constants in the doped state, denoted as $J_{d}$, compared to the undoped state, *J*. The ionic radii of Nd^3+^, Sm^3+^, and La^3+^ are 1.27 $\dot{A}$, 1.24 $\dot{A}$, and 1.363 $\dot{A}$, respectively, all of which are smaller than that of Sr^2+^ (1.44 $\dot{A}$) [24]. As a result, a compressive strain emerges, leading to a reduction in the lattice parameters with increasing RE dopant concentrations. This behavior has also been reported by Blasco et al. [35] and Michalik et al. [24] for Nd, Sm, and La ion doping. Our findings indicate that this decrease in lattice parameters modifies the spin exchange interactions, leading to a weakening of the Cr-O-Re coupling. A similar reduction in this coupling has been reported by Blasco et al. [35], which was attributed to an increase in Cr-O-Cr pathways. In our model, this implies that the anti-site disorder rises when increasing the RE concentration *x*, which in turn enhances the antiferromagnetic exchange interaction constant in the doped system compared to that in the undoped state, $J_{d}^{Cr-Re}<J^{Cr-Re}$. Notably, the bond lengths of Re-O and Cr-O remain nearly unchanged with increasing RE doping concentration [5]. With the increase in $J_{d}^{Cr-Re}$ and the rise in anti-site disorder, anti-site defects are the primary factors contributing to the reduction in magnetization *M*. Moreover, the substitution of higher valance ions in place of Sr^2+^ results in electron doping at the Re sites in the system which leads to an enhanced magnetic moment of Re. From Equation (13), it can be seen that this leads to a decrease in the total magnetization *M* in the doped SCRO compound. The results for Nd-, Sm-, and La-doped SCRO are presented in Figure 4 for doping concentrations of *x* = 0–0.2, where we assume that no clusters and secondary phases of the doping ions are formed. These results show good qualitative agreement with the experimental data reported by Blasco et al. [35] and Michalik et al. [24,25]. It is important to note that under tensile strain, where the lattice parameters increase, an enhancement in magnetization would be expected. Using ab initio calculations, Ain et al. [36] observed such behavior in SCRO under two types of tensile strain: biaxial and hydrostatic.

Furthermore, it should be emphasized that the coercive field $H_{c}$ increases with increasing Nd or Sm doping concentrations. A similar increase in $H_{c}$ has been reported in BFRO by Escanhoela et al. [37], where the pressure was varied from 1.5 GPa to 22 GPa.

The Curie temperature $T_{C}$ exhibits a slight decrease with increasing Nd doping in SCRO (see the inset in Figure 4). It is found that substitution of Sr^2+^ by Nd^3+^ reduces the $T_{C}$ of SCRO [24,35]. This observation, along with the magnetic behavior associated with RE ion doping, can be utilized in the development of new and more efficient materials for spintronic devices.

Notably, it has been reported that electron doping in Sr_2_FeMoO_6_ double perovskites, achieved by replacing divalent Sr^+2^ with trivalent La^+3^ [38] and Nd^+3^ [39], results in an increase in the Curie temperature. Specifically, a 70 K increase is observed for 50% La doping [38], while 40% Nd doping leads to a 90 K increase in $T_{C}$ [39]. The properties of ion-doped Mo-based double perovskites will be examined in a subsequent study.

Similarly, a decrease in magnetization *M* is obtained for La-doped BFRO (see Figure 4, curve 4), which is consistent with the experimental findings of Wang et al. [27]. The underlying mechanism is analogous to that observed in SCRO. The antiferromagnetic exchange interaction parameter increases with La doping, such that $J_{d}^{Fe-Re}<J^{Fe-Re}$. The doped electrons are predominantly injected into the Re 5d states [27]. As a result, the magnetic moment of Re increases with higher La concentrations, and according to Equation (10), the total magnetization *M* decreases.

### 3.3. Temperature, Size, and Doping Dependence of the Band Gap Energy in BFRO and SCRO

**Figure 5:**
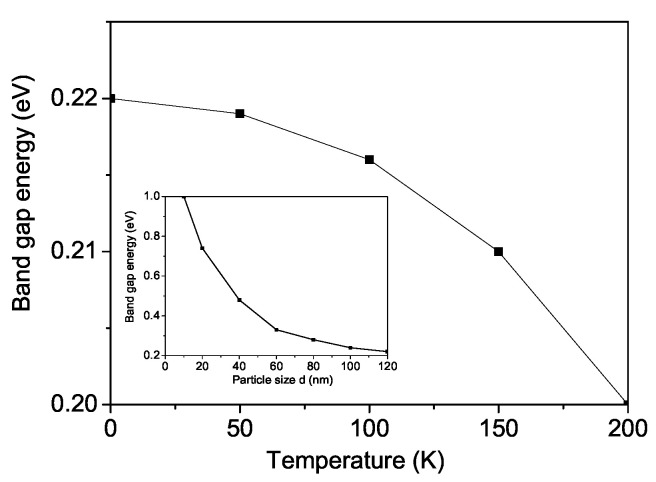
Temperature dependence of the band gap energy $E_{g}$ for bulk SCRO. Inset: Particle size dependence of the band gap energy $E_{g}$ for SCRO.

From Equations (5) and (6), we have computed the band gap energy $E_{g}$ in SCRO. Temperature has a significant impact on the band gap $E_{g}$ of a material, and the dependence of $E_{g}\left(T\right)$ is illustrated in Figure 5. The results indicate that $E_{g}$ decreases with increasing temperature, a trend that was also observed in BFRO. Figure 5 proves the adequacy of the presented model and is in agreement with the experimental results and Equation (6). The latter determines the influence of the magnetic ordering on $E_{g}$, which decreases with increasing temperature due to a decrease in the magnetization (see Figure 1). The nonlinear character of M(T) also determines the nonlinear nature of $E_{g}\left(T\right)$.

Due to size and surface effects, the magnetization *M* decreases, while $E_{g}$ increases as the NP size decreases (see inset in Figure 5). The nonlinear nature of M(d) (see Figure 2), in combination with Equation (6), determines the nonlinear behavior of $E_{g}\left(d\right)$, i.e., a reduction in the surface-to-volume ratio through the intra-atomic interaction leads to a rise in the top of the valence band and a lowering to the bottom of the conduction band, and to a reduction in the band gap width. This behavior is consistent with the experimental findings of Singh et al. [40]. Leng et al. [41] reported an optical band gap value of 1.39 eV in Sr_2_FeReO_6_ powders.

**Figure 6:**
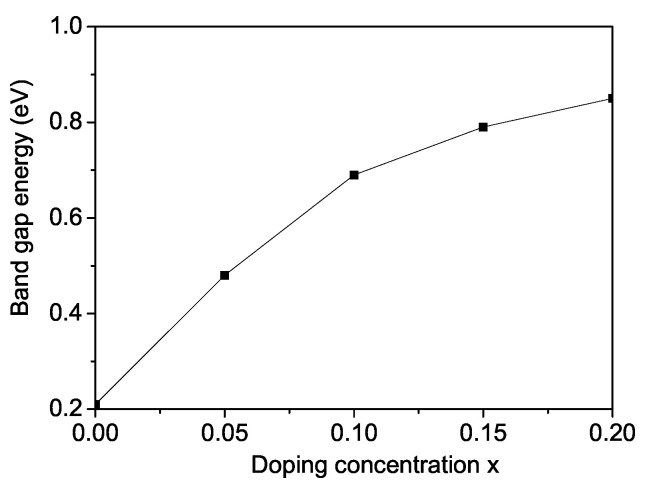
Nd doping concentration dependence of the band gap energy for SCRO at *T* = 150 K.

Because of the direct influence of the band gap on optoelectronic characteristics, band gap adjustment in perovskite materials is crucial. It specifies the range of light wavelengths that a substance may absorb and convert into an electrical signal. The ability to modify the band gap from a low range to a high range has made perovskites appealing for a diversity of applications including photovoltaics, lasing, light-emitting devices, photodetectors, high energy, and particle detection. The semiconducting materials with lower band gaps are essential for capturing more of the visible solar spectrum. When substituting Sr in SCRO with RE ions, a compressive strain develops, leading to the formation of anti-site defects, an increase in Cr/Re disorder, and an enhancement of the antiferromagnetic exchange interaction between Cr and Re ions. Moreover, it should be emphasized that the exchange interaction constant $J_{ij}=J\left(r_{i}-r_{j}\right)$ depends on the distance between the spins, on the lattice parameters, and on the lattice symmetry. As a result, the band gap energy $E_{g}$ increases with increasing ion doping concentration *x*. The dependence of $E_{g}$ on the Nd doping concentration *x* in bulk SCRO is shown in Figure 6. It should be noted that the substitution of doping ions creates an impurity –*d* zone near the valence band. As the concentration of Nd increases, the magnetization decreases (see Figure 4, curve 1), which will lead to a conduction zone shifting upwards and shifting the top of the valence band downwards, i.e., to an increase in $E_{g}$. A similar effect of ion doping is also observed in BFRO. An enhancement of the band gap energy $E_{g}$ has been reported by Quattropani et al. [42] for Bi_2_FeCrO_6_ double-perovskite thin films.

### 3.4. Temperature Dependence of the Phonon Energy and Damping in BFRO and SCRO

Raman spectroscopy employs the Raman effect for substances analysis. Raman spectroscopy is used to analyze a wide range of materials, including gases, liquids, and solids. Highly complex materials such as biological organisms and human tissue can also be analyzed by Raman spectroscopy. For solid materials, Raman scattering is used as a tool to detect high-frequency phonon and magnon excitations.

The spin–phonon interaction from a microscopic point of view is related to the modulation of the distance between neighboring spins by polar modes. This modulation leads to a change in the exchange interaction. The spin–phonon interaction is accounted for by the development of the exchange interaction according to the displacements of the magnetic ions with regard to their equilibrium position up to the second order. From a microscopic point of view, this means that the exchange interactions become temperature-dependent (see $J_{eff}$, page 8).

As concerns the microscopic model, we think that, in this way, the influence of the spin–phonon interaction on the properties of the materials is taken into account (note that Equations (18) and (19) give the dependence of phonon frequencies on the magnetic ordering), i.e., we have a self-consistent influence between the magnetic and lattice subsystems.

Finally, we will study the temperature dependence of the phonon energy ω and damping γ of the A_1*g*_ mode of BFRO, at $\omega_{0}$ ∼ 603 cm^−1^. As mentioned above, we found an analytical expression for the phonon excitation of the BRFO material (see Equation (18)). The phonon spectrum is determined by the temperature *T*, the magnetization *M*, the anharmonic spin–phonon coupling parameter *R*, and the anharmonic phonon interactions *A* and *B*. The following discussion presents our results and compares them with the existing experimental data. The sign of the spin–phonon interaction constant *R* determines whether the phonon frequencies experience hardening or softening below the critical temperature [43]. The specific outcome depends on the interaction between the subsystems and is thus a material-specific property.

**Figure 7:**
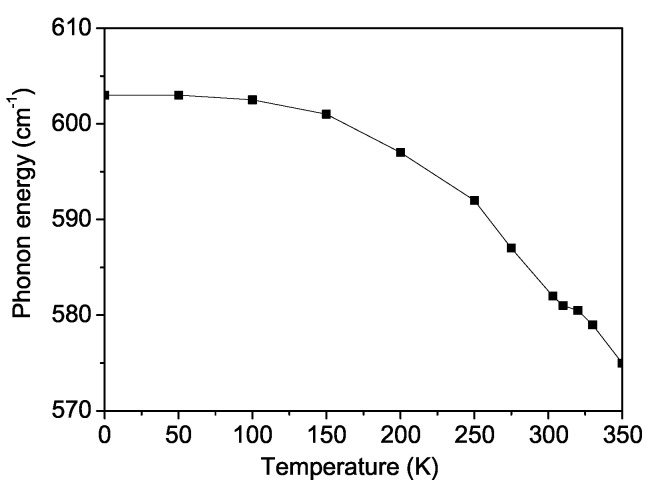
Temperature dependence of the phonon energy of the phonon A_1*g*_ mode at $\omega_{0}$ = 603 cm^−1^ for BFRO.

Figure 7 depicts this A_1*g*_ phonon mode at $\omega_{0}$ ∼ 603 cm^−1^ as a function of the temperature, where the phonon energy ω, corresponding to the Raman line position, decreases by approximately 25 cm^−1^ below the magnetic-phase-transition temperature $T_{C}$. Additionally, near the Curie temperature of $T_{C}$ = 300 K, an anomaly is observed in the temperature dependence of the phonon energy ω(T), providing strong evidence of significant spin–phonon interaction in BFRO, as reported experimentally by Garcia-Flores et al. [28]. The phonon properties are highly sensitive to the anharmonic spin–phonon interaction parameter *R*. It should be noted that the appearance of a kink around $T_{C}$ is related to the fact that, above the phase transition point, the temperature dependence of the phonon mode is determined only by anharmonic phonon–phonon interactions, while below $T_{C}$, with a decrease in temperature, the spin–phonon interaction begins to play a major role. Below $T_{C}$, the nonlinear nature of the curves is due to the fact that the phonon frequency depends on the square of the magnetization $M^{2}$, which also explains the saturation at very low temperatures.

**Figure 8:**
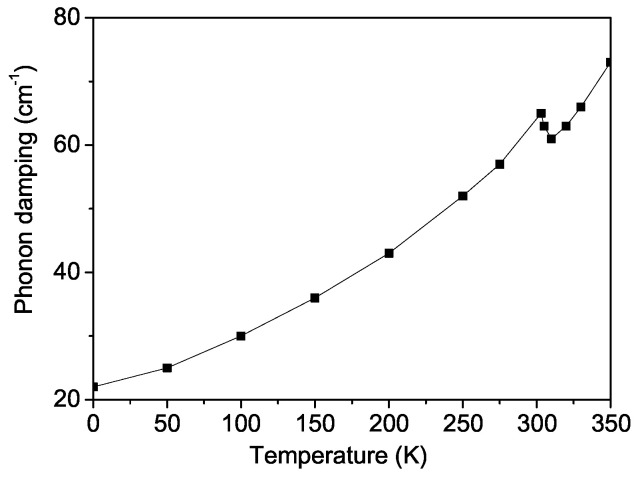
Temperature dependence of the damping of the phonon A_1*g*_ mode at $\omega_{0}$ = 603 cm^−1^ for BFRO.

Figure 8 illustrates the temperature dependence of the phonon damping γ(T), which corresponds to the Raman line width. The damping γ increases strongly with rising temperature *T* and exhibits a distinct anomaly near the Curie temperature $T_{C}$. The kink around $T_{C}$ in the damping curve (shown in Figure 8) is determined by the fact that above $T_{C}$, damping occurs only due to phonon–phonon scattering, while below $T_{C}$, phonon damping occurs through the creation of spin deviations ($\langle S^{-}S^{+}\rangle$). The observed temperature dependence of both the phonon energy and damping for the $\omega_{0}∼$ 603 cm^−1^ mode in BFRO is in good qualitative agreement with the experimental findings of Garcia-Flores et al. [28] for BFRO.

A similar behavior is observed for the A_1*g*_ phonon mode $\omega_{0}$ ∼ at 622 cm^−1^ in SCRO, where an anomaly appears at the Curie temperature of $T_{C}$ = 635 K. This result is consistent with the experimental data reported by Garcia-Flores et al. [28] for SCRO.

## 4. Conclusions

In conclusion, we have explored the magnetic, optical, and phonon properties of both pure and ion-doped BFRO and SCRO, considering bulk materials as well as NPs, through a microscopic model and Green’s function theory. Our results show that as the NP size decreases, both the magnetization *M* and the Curie temperature $T_{C}$ also decrease.

Substituting Ba or Sr ions with Sm, Nd, or La ions induces a compressive strain due to the smaller ionic radii of the dopants compared to the host ions. This strain leads to changes in exchange interactions, enhanced antiferromagnetic superexchange interactions between Fe or Cr ions and Re ions, and increased magnetic moments of Re. As a result, the magnetization *M* decreases with increasing dopant concentration *x*.

We have also examined the temperature, size, and ion doping dependence of the band gap energy $E_{g}$ in SCRO. Additionally, we calculated the temperature dependence of the phonon energy ω and damping γ of the A_1*g*_ mode in BFRO and SCRO. An anomaly around the Curie temperature $T_{C}$ was observed, providing strong evidence of a significant spin–phonon interaction in these materials. It should be emphasized that the electron–phonon interaction also plays an important role in the phonon properties of double perovskites which will be studied in future research.

## Data Availability

Data sharing not applicable to this article as no data sets were generated or analysed during the current study.
